# Effects of Electron Irradiation and Temperature on Mechanical Properties of Polyimide Film

**DOI:** 10.3390/polym15183805

**Published:** 2023-09-18

**Authors:** Jian Qiu, Jusha Ma, Wenjia Han, Xiao Wang, Xunchun Wang, Maliya Heini, Bingyang Li, Dongyang Sun, Ruifeng Zhang, Yan Shi, Cunfa Gao

**Affiliations:** 1 State Key Laboratory of Mechanics and Control for Aerospace Structures, Nanjing University of Aeronautics and Astronautics, Nanjing 210016, China; swordqiu@163.com (J.Q.); xiaowang@nuaa.edu.cn (X.W.); cfgao@nuaa.edu.cn (C.G.); 2 School of Chemistry and Civil Engineering, Shaoguan University, Shaoguan 512005, China; 3 Shanghai Institute of Space Power-Sources, Shanghai 200245, China; jushama@163.com (J.M.); hanbeihang@163.com (W.H.); runze0916@126.com (X.W.); 4 Xinjiang Key Laboratory of Electronic Information Materials and Devices, Chinese Academy of Sciences, Urumqi 830011, China; maliya@ms.xjb.ac.cn; 5 China Academy of Aerospace Science and Innovation, Beijing 100871, China; 6 College of Engineering, Peking University, Beijing 100871, China; 7 School of Naval Architecture and Ocean Engineering, Jiangsu University of Science and Technology, Zhenjiang 212003, China; sundongyang@cqu.edu.cn; 8 School of Electrical Information Engineering, Ningxia Institute of Science and Technology, Shizuishan 753000, China; zhangruifeng@nuaa.edu.cn

**Keywords:** electron irradiation, high and low temperature, polyimide film, mechanical property

## Abstract

Polyimide (PI) is widely deployed in space missions due to its good radiation resistance and durability. The influences from radiation and harsh temperatures should be carefully evaluated during the long-term service life. In the current work, the coupled thermal and radiation effects on the mechanical properties of PI samples were quantitatively investigated via experiments. At first, various PI specimens were prepared, and electron irradiation tests were conducted with different fluences. Then, both uniaxial tensile tests at room temperature and the dynamic mechanical analysis at varied temperatures of PI specimens with and without electron irradiation were performed. After that, uniaxial tensile tests at low and high temperatures were performed. The fracture surface of the PI film was observed using a scanning electron microscope, and its surface topography was measured using atomic force microscopy. In the meantime, the Fourier-transform infrared spectrum tests were conducted to check for chemical changes. In conclusion, the tensile tests showed that electron irradiation has a negligible effect during the linear stretching period but significantly impacts the hardening stage and elongation at break. Moreover, electron irradiation slightly influences the thermal properties of PI according to the differential scanning calorimetry results. However, both high and low temperatures dramatically affect the elastic modulus and elongation at break of PI.

## 1. Introduction

Due to its good mechanical properties and lightweight, a polyimide (PI) film is usually applied as a substrate material of space solar cells and even structural material in spacecraft. Recently, a PI-based flexible solar wing was successfully deployed in the core module assembly of China Space Station for the first time [1]. The materials and structures employed in space missions will endure unfavorable irradiation and alternate temperatures, which usually affect their mechanical properties [2,3]. Considering the radiation effect, the National Aeronautics and Space Administration (NASA) has taken all kinds of PI films into the International Space Station (ISS) to assess their durability via the Materials International Space Station Experiment (MISSE). Many achievements have been reported [4,5,6,7]. After one to two years of space exposure in a low Earth orbit (LEO), the PI specimens show degradation in surface characteristics and mechanical properties. However, the results from ground tests show that electron beam (EB) and ultraviolet (UV) irradiation had an insignificant impact on its mechanical properties. It is reported that the erosion from atomic oxygen (AO) irradiation in a LEO causes the degradation of the mechanical properties of PI films [8,9]. Ground tests rather than in-orbit experiments are more applicable for cost and time-saving. Recent research reveals that an irradiation-load coupling effect appeared by applying high tensile stress to PI during electron irradiation on the ground. The radiation damage effect of polyimide fibers under electron irradiation and gamma-ray radiation and the coupling effect of irradiation with preload are presented [10,11,12]. Electron irradiations were reported to have considerable influence on the mechanical properties of other components, such as polymers [13,14,15] and semiconductor materials [16]. In summary, high-energy electron irradiation in space has a noticeable effect on PI’s morphology and mechanical properties [17].

Moreover, PI will experience harsh high- and low-temperature cycles in space. The risk of thermal stress concentration may occur after a thermal cycling test to solar array with flexible printed circuit (FPC), whose main structure is made of PI, with temperatures ranging from −100 °C to 130 °C [18]. Similar fracture behaviors have been observed for thin-film solar cells after thermal cycling and thermo-vacuum tests [19]. Furthermore, the tensile strength, stretching modulus, and elongation variations in polyimide films after temperature alternating from −190 °C to +200 °C have also been reported [20]. The stability of PI under electron irradiation can be attributed to the aromatic imide ring structure [21]. From the DMA and DSC analyses [22], there exists a decrease in the thermal stability of irradiated PI. The tensile properties of PI are hardly affected by electron irradiation, and the formation of crosslinking results in a change in glass transition temperature observed in irradiated polyimides [23,24]. Under the joint effect of 2 MeV electron irradiation and high temperature at 373 K, mechanical tests showed an increase in plasticity, and long-term hardening was observed [25]. The effects of low temperature and mechanical loading were seldom investigated.

Systematic research on the mechanical behaviors of a PI film under coupling effects of electron irradiation and harsh temperature is presented in this work. The PI specimens were first irradiated by high-energy electrons. Then, quasi-static, dynamic stretching, and high- and low-temperature tests were carried out. At last, various characterization experiments were performed.

## 2. Materials and Methods

**Figure 1:**
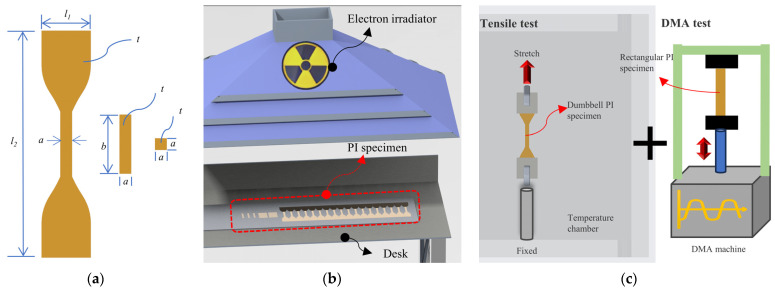
Workflow of this study. (**a**) PI specimens of different shapes. (**b**) Schematic illustration of electron irradiation. (**c**) Uniaxial tensile test and DMA test.

The workflow of this study is summarized in Figure 1. At first, all the PI specimens including dumbbell-shaped, rectangular, and square tensile test specimens were cut by a silhouette CAMEO desktop cutting machine (see Figure 1a). Moreover, specimens for mechanical tests were fabricated along the same axial direction. Then, some PI specimens were subjected to an electron irradiation test (see Figure 1b). After that, uniaxial tensile and DMA tests were performed to evaluate the mechanical properties of the PI specimens with or without electron irradiation, and variable temperature factors were also considered (see Figure 1c).

More specific information about the test type and quantity of the PI specimens is listed in Table 1. All the PI specimens were double-checked under an optical microscope to eliminate samples with unexpected flaws.

Electron irradiation experiments were conducted at Xinjiang Technical Institute of Physics & Chemistry, Chinese Academy of Sciences. The ELV-8 II electron accelerator can provide 1 MeV electron beam with an electron flux of 1.012 × 10^12^ e/(cm^2^·s). According to ISO standard 23038 [26] and China national standard GB 38190 [27], the PI specimens were exposed to a maximum total fluence of 1 × 10^16^ e/cm^2^. Details are shown in Table 2.

Uniaxial tensile tests were carried out by a universal material testing machine (Range 500 N, Instron 5943, Boston, MA, USA). PI specimens both with and without electron irradiation were elongated to breakage. The loading speed was set as 100 mm/min, as suggested by China national standard GB/T 1040.3 [28], at ambient temperature and humidity. The whole loading process was recorded by a high-resolution CCD camera.

To investigate the high and low temperature effects, tensile tests with different temperatures were carried out in a temperature chamber. The testing temperature was increased or decreased from room temperature at a constant rate of 10 °C/min to a specific value and then was maintained for about 30 min. Based on the working temperature in space, high temperatures were chosen from 60 °C to 200 °C at intervals of 20 °C, and low temperatures were chosen from 0 °C to −90 °C at intervals of 30 °C.

The dynamic mechanical analysis (DMA) test of PI was carried out by TA DMA850 to investigate the coupling effect of temperature and electron irradiation. Rectangular PI specimens and the thin-film tensile kit were selected to perform the tensile test in the atmosphere. At first, the temperature was increased from room temperature to about 390 °C at a speed of 5 °C/min. Then, a new set of specimens fabricated from the same slice was tested as temperature decreased from room temperature to about −90 °C at a speed of 5 °C/min. The frequency was 1 Hz, and the amplitude was 20 microns.

The influence of electron irradiation on the glass transition temperature (Tg) was measured by a differential scanning calorimetry (DSC) instrument (TA Q2000, New Castle, DE, USA). The temperature ranged from room temperature to 500 °C in a nitrogen environment, at a rate of 5 °C/min. Moreover, the comparative group was cooled down to −120 °C rapidly, then heated up to 20 °C at a rate of 5 °C/min by TA DSC250. The fracture surface of PI after the tensile test was observed using scanning electron microscopy (SEM, FEI Quanta 650, Columbus, OH, USA). The surface topography was measured using atomic force microscopy (AFM, Oxford Instruments Asylum Research Cypher ES, San Diego, CA, USA) in a tapping mode. Firstly, an area of 5 μm × 5 μm on a PI specimen was randomly selected to perform the coarse scan, and then, an area of 1 μm × 1 μm within was selected to perform the fine scan. Fourier-transform infrared spectroscopy (FTIR) test was carried out using ThermoFisher Nicolet iS20 (Waltham, MA, USA). The wavenumber was chosen from 400 to 4000, and a transmittance mode was selected to perform the test. All the above specimens were cleaned with absolute alcohol and deionized water before the test.

## 3. Results

### 3.1. Uniaxial Tensile Test

#### 3.1.1. Effect of Electron Irradiation

**Figure 2:**
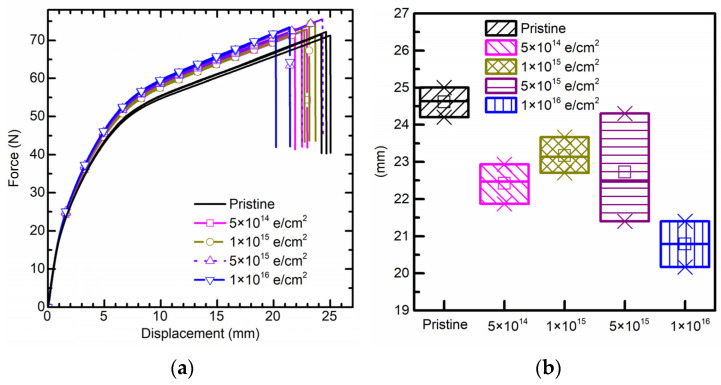
(**a**) Force–displacement curve from uniaxial tensile test for polyimide specimens with none, 5 × 10^14^ e/cm^2^, 1 × 10^15^ e/cm^2^, 5 × 10^15^ e/cm^2^, and 1 × 10^16^ e/cm^2^ electron irradiation fluence. (**b**) Maximum elongation at break.

Based on the force–displacement curves of the uniaxial tensile test (Figure 2a), electron irradiation has negligible influence on the elastic modulus of PI in the linear elastic stage. However, in the hardening stage, PI becomes “stronger” after electron irradiation and is gradually reinforced by the radiation fluence. Moreover, the secant modulus of PI in the hardening stage shows an increasing trend with electron irradiation.

Furthermore, electron irradiation significantly affects elongation at break of PI. When the total electron fluence reaches 5 × 10^14^ e/cm^2^, the mean elongation at break decreases by 8.9%. When electron fluence reaches a maximum of 1 × 10^16^ e/cm^2^, the mean elongation at break decreases by 15.5% (Figure 2b). On the contrary, the ultimate tensile strength tends to increase after electron irradiation.

**Figure A1:**
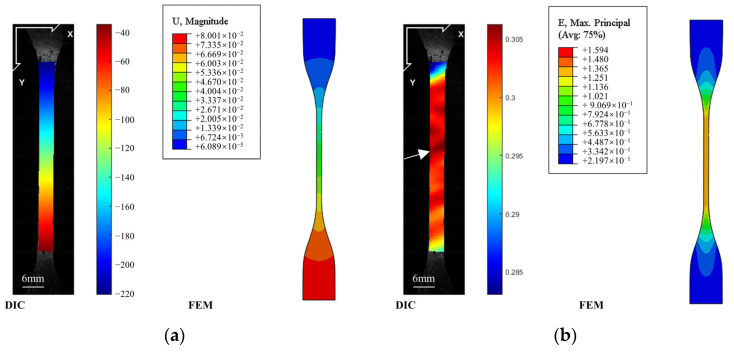
DIC results and FEM simulation. (**a**) Displacement. (**b**) Strain.

Amorphous regions and semi-crystalline regions coexist and overlap each other in PI material. The latter is related to mechanical properties. The elastic stage is dominated by the elongation of semi-crystalline and amorphous regions, which cross each other and are insensitive to electron irradiation. However, the hardening stage is dominated by the alignment and sliding of the crystalline region, where electron irradiation may cause chemical changes and affect the crystalline phase. Finally, the electron irradiation may bring reinforcement and embrittlement to the PI specimen. As a result, the ultimate tensile strength of PI film increases, but elongation at break decreases after irradiation. Finally, the tensile process of PI was further evaluated by digital image correlation (DIC) algorithm, more details can be seen in Figure A1 of Appendix A.

#### 3.1.2. Effect of High Temperature

**Figure 3:**
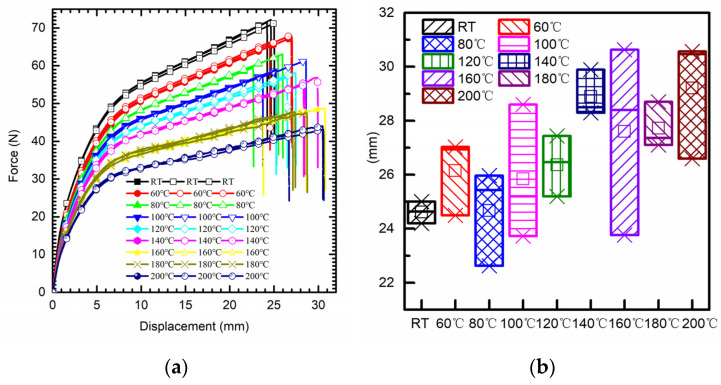
(**a**) Force–displacement curve from uniaxial tensile test with high temperature. (**b**) Maximum elongation at break.

As shown in Figure 3a, the high temperature of the tensile test chamber was set from 60 °C to 200 °C, with increments of 20 °C for each tensile set. Compared to the tensile results at room temperature, the mean break elongation shows an increasing trend with the rise in temperature. In contrast, elastic modulus and tensile strength present an opposite trend. Compared to the elongation at break at room temperature, an increase of 12.1% and 18.7% were observed at the temperatures of 160 °C and 200 °C, respectively (see Figure 3b). Regarding the ultimate tensile strength, a decrease of 34% and 39.9% appeared at the temperatures of 160 °C and 200 °C, respectively. The mean elastic modulus at room temperature was about 2.45 GPa, which decreased to 1.58 GPa and 1.43 GPa at the temperatures of 160 °C and 200 °C, respectively.

#### 3.1.3. Effect of Low Temperature

**Figure 4:**
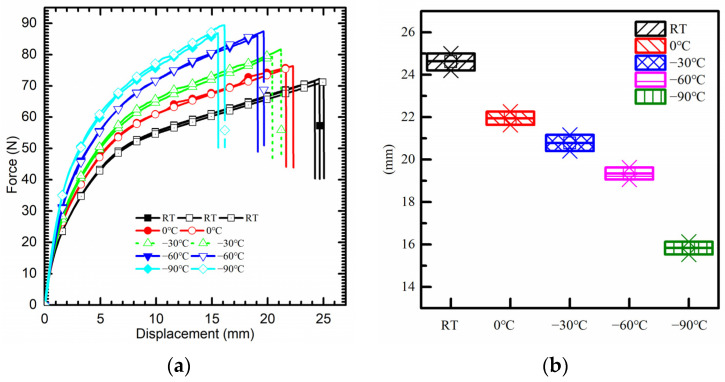
(**a**) Force–displacement curve from uniaxial tensile test with low temperature. (**b**) Maximum elongation at break.

The low temperature range was set from 0 °C to −90 °C, with decrements of 30 °C for each tensile set as shown in Figure 4a. Similar to the cases in high temperatures, the mechanical properties of PI show an enhancement trend with the decrease in temperature. Compared to the ultimate tensile strength at room temperature, a maximum increase of 23% was achieved at the temperature of −90 °C. Moreover, the secant modulus of PI in the hardening stage presents an increase of 77.8%. However, a decrease in mean elongation at break comes to 35.7% at the temperature of −90 °C compared to that at room temperature (see Figure 4b).

### 3.2. DMA Test

#### 3.2.1. Effect of Electron Irradiation and High Temperature

**Figure 5:**
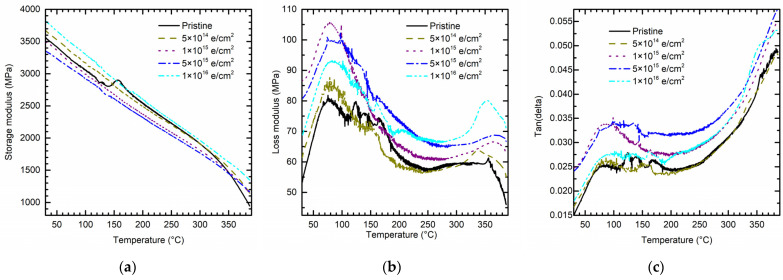
DMA test from 30 °C to 390 °C. (**a**) Storage modulus. (**b**) Loss modulus. (**c**) Phase angle (damping factor).

The storage modulus of PI with or without electron irradiation presents a kind of decrease as the temperature increases from 30 °C to 390 °C (Figure 5a), which exhibits a “softening” process. However, this trend shows a bifurcation when the temperature comes to about 350 °C. The storage modulus for the samples without electron irradiation appears to drop rapidly, while for those with irradiation, it appears to be smaller. That means electron irradiation may influence the glass transition temperature (Tg), which was validated in the following DSC test.

From the loss modulus curve (Figure 5b), PI with or without electron irradiation can be divided into three to four stages. When the temperature increases from 30 °C to about 90 °C, the loss modulus of PI presents an increasing trend. However, when the temperature is over 90 °C, the loss modulus slope shows a kind of oscillation and a decreasing trend. When the temperature is more than 250 °C, the loss modulus slope shows a kind of smoothness. Finally, when the temperature is over 350 °C, there is a clear bifurcation in loss modulus curve of PI before and after electron irradiation.

Similarly, the damping factor δ (see Figure 5c) also undergoes three to four dissimilar stages as the temperature changes, but in a different trend. When the temperature is more than 300 °C, the damping curve presents an opposite trend compared to the loss modulus (Figure 5b), that is, because the storage modulus shows a decline after the temperature is higher than 300 °C, as shown in Figure 5a.

According to previous research [11,12], the loss modulus at around 250 °C depends on the transitions due to the rotation of aromatic rings (β-transition). The loss modulus slope around 250 °C shows no significant difference with or without electron irradiation, which may indicate that the structure of aromatic rings was not influenced by electron irradiation. When the temperature is over 350 °C, the loss modulus is related to the segmental motion of the backbone (α-transition). Combined with the storage modulus results, it can be concluded that electron irradiation brings the scission of chemical bonds in PI and forms radicals. This may cause molecular chain crosslinking and therefore change the PI’s crystalline state.

#### 3.2.2. Effect of Electron Irradiation and Low Temperature

**Figure 6:**
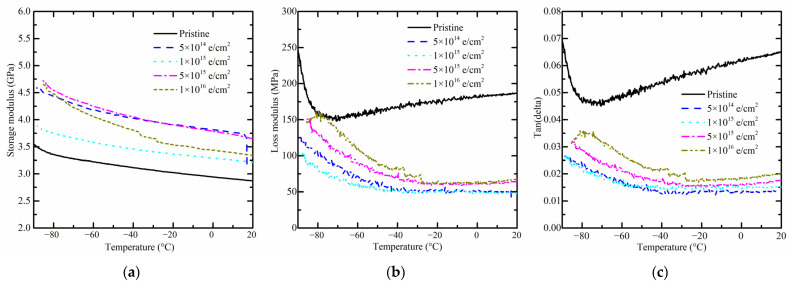
DMA test from 20 °C to −90 °C. (**a**) Storage modulus. (**b**) Loss modulus. (**c**) Phase angle (damping factor).

As the temperature decreases from 20 °C to −90 °C, the storage moduli of PI with or without electron irradiation all present a kind of increase, which exhibits a “hardening” process (see Figure 6a). After receiving a total electron radiation fluence of 1 × 10^16^ e/cm^2^, the storage modulus of PI presents a most significant increase as the temperature approaches −90 °C. Meantime, a similar phenomenon can be observed in the case of loss modulus (see Figure 6b) and damping factor (see Figure 6c). Unlike the high temperature situation, storage modulus, loss modulus, and damping factor all present an ascending trend as the temperature descends. Referring to the results of Section 3.1.1, electron irradiation had an insignificant effect on the elastic stage of PI, when quasi-static tests were carried out at ambient temperature, while the DMA test focused on small displacement in a cyclic loading process. Therefore, under the dynamic test condition, there is a coupling effect of electron irradiation and low temperature on the mechanical behavior of PI.

### 3.3. Changes in Thermal Properties

**Figure 7:**
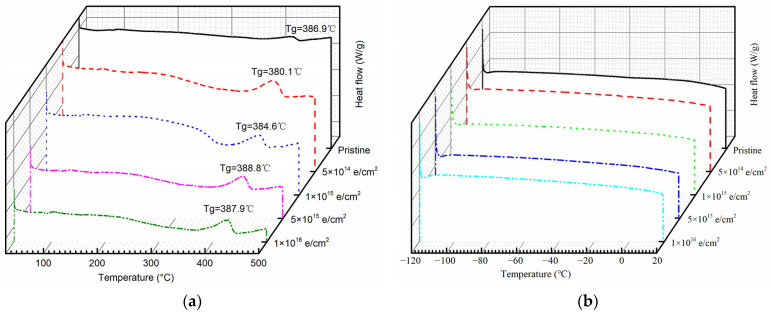
DSC curve of pristine PI samples and those after receiving radiation fluence of 5 × 10^14^ e/cm^2^, 1 × 10^15^ e/cm^2^, 5 × 10^15^ e/cm^2^, and 1 × 10^16^ e/cm^2^. (**a**) Room temperature to 500 °C; (**b**) −120 °C to room temperature.

DSC tests were carried out to investigate whether electron irradiation impacts thermal properties of PI, such as glass transition temperature (Tg). Firstly, a small bump around 100 °C is observed, and the glass transition temperature fluctuates between 380 °C and 390 °C. After receiving a total fluence of 5 × 10^15^ e/cm^2^, the Tg temperature reaches a maximum of 388.8 °C (Figure 7a). Then, as the temperature rises from −90 °C to 20 °C, the DSC curve presents a slowly ascending slope both with and without electron irradiation (Figure 7b). From the above DSC results, the thermal properties of PI are almost unaffected by electron irradiation. From the reference, electron irradiation leads to crosslinking of the polyimide chains and may induce a higher glass transition temperature [29]. As a result, crosslinking of polyimide chains is not likely to happen when receiving a low electron fluence such as 5 × 10^14^ e/cm^2^.

### 3.4. Changes in Morphology

#### 3.4.1. Morphology Changes on Fracture Surface

**Figure 8:**
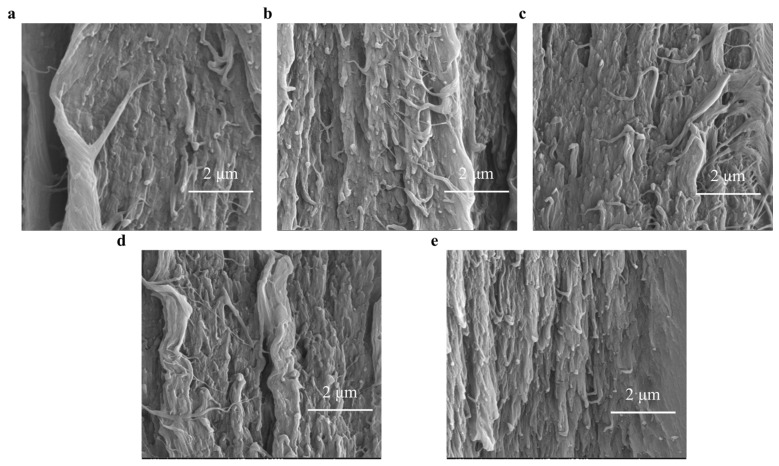
SEM images. (**a**) Fracture surface of pristine polyimide film after tensile test. (**b**) Fracture surface of polyimide film after receiving radiation fluence of 5 × 10^14^ e/cm^2^, (**c**) 1 × 10^15^ e/cm^2^, (**d**) 5 × 10^15^ e/cm^2^, and (**e**) 1 × 10^16^ e/cm^2^.

After the tensile test, the fracture surfaces of the PI specimens were studied under SEM. As shown in Figure 8, when down to the scale of microns, the inner space of PI is not entirely intact and spaceless. The morphology of fracture section presents a layered structure full of microvoids because some gas was produced during electron radiation [30]. The inner structure may change topography during electron irradiation and other “defects” are generated [31]. Then, the aggregation and entanglement of the “defects” chains changed the matrix topology. Another noticeable topology change is that more fiber-like structures appear after a different fluence of electron irradiation, as shown in Figure 8b–e.

#### 3.4.2. Morphology Changes on the Surface

**Figure 9:**
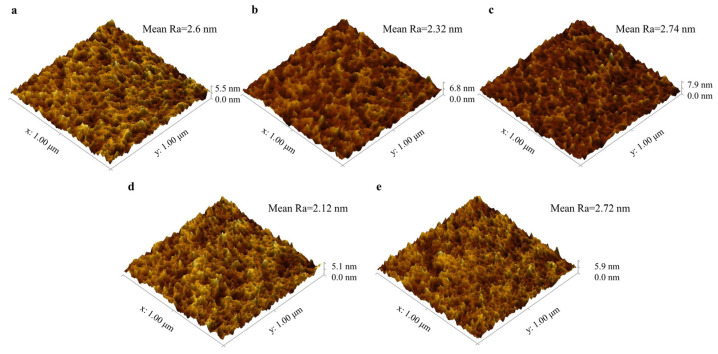
AFM testing. Three-dimensional surface topography of (**a**) pristine polyimide film and those after receiving radiation fluence of (**b**) 5 × 10^14^ e/cm^2^, (**c**) 1 × 10^15^ e/cm^2^, (**d**) 5 × 10^15^ e/cm^2^, and (**e**) 1 × 10^16^ e/cm^2^.

Furthermore, the surface topography of the specimens was measured by AFM. From previous research, the surface roughness of PI may either increase significantly [11] or decrease after electron irradiation [32]. According to our research, the variations of the surface roughness are nonmonotonic. As shown in Figure 9, the average roughness of pristine PI is 2.603 nm and drops to 2.318 nm after receiving electron irradiation of 5 × 10^14^ e/cm^2^. Then, it reaches a maximum of 2.737 nm as irradiation fluence increases to 1 × 10^15^ e/cm^2^. Further, it comes to a minimum of 2.118 nm in the case of 5 × 10^15^ e/cm^2^ and increases to 2.716 nm after irradiation fluence of 1 × 10^16^ e/cm^2^. More details about measured roughness are listed in Table A1 of Appendix A, and other influencing factors are discussed. Due to previous experimental data [33], 1 MeV electron has sufficient energy to penetrate through the 50-micron thickness of the PI specimens employed in this study. Apparent morphology changes can be observed both on the interior and exterior surfaces.

## 4. Discussion and Conclusions

**Figure A2:**
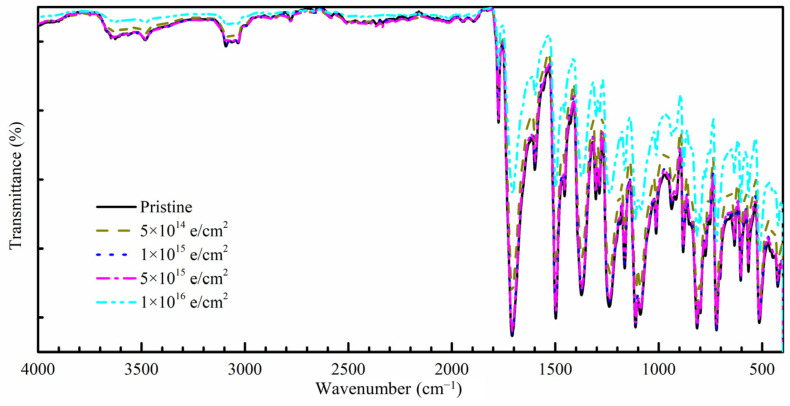
FTIR curve of pristine PI samples and those after receiving radiation fluence of 5 × 10^14^ e/cm^2^, 1 × 10^15^ e/cm^2^, 5 × 10^15^ e/cm^2^, and 1 × 10^16^ e/cm^2^.

**Figure A3:**
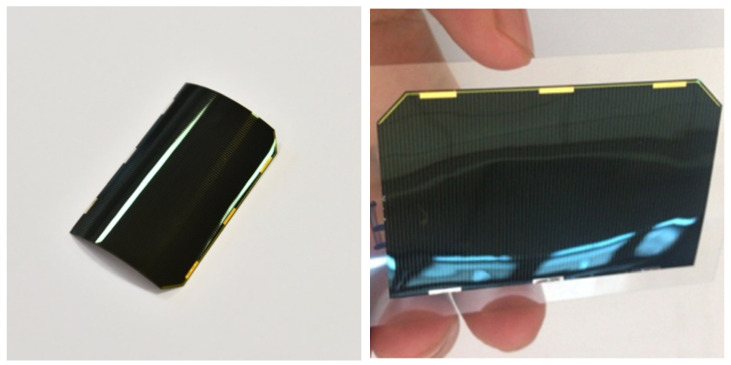
Thin-film solar cell based on PI film.

Based on the FTIR test results, no new substance is created after electron irradiation. However, significant differences on each peak of the pristine and irradiated PI specimens indicate a change in the quantity of each chemical composition. More details can be seen in Figure A2 and Table A2 of Appendix A. From the tensile test results after electron irradiation, electron irradiation has little influence on mechanical properties in the linear elastic stage. An increase in stretching stiffness and decrease in elongation at break can be observed during the nonlinear stretching stage, which is believed to be induced by electron irradiation. Regarding the temperature effect on the mechanical properties of PI, high temperature causes a decline in elastic modulus but an increase in elongation at break. The opposite trend is observed in the case of low temperatures. Changes in thermal properties can be attributed to molecular crosslinking and chain scission. In this study, electron irradiation had negligible influence on the thermal stability of the PI samples. The rotation of aromatic rings (β-transition) of PI is not likely influenced by electron irradiation, but the segmental motion of the backbone (α-transition) of PI is slightly changed. Both surface and inner topography of the PI samples changes with electron irradiation in a complex way. The aggregation and entanglement of molecular chains as well as generation of internal gas may be the reasons; both bring adverse influences on PI-based flexible space devices such as thin-film solar cells (see Figure A3 in Appendix A). Electron irradiation and low temperature have a coupling effect on PI under dynamic test conditions, which should be thoroughly investigated in the future.

## Figures and Tables

**Table 1:** List of PI samples.

Shape/Type	Test Type	Dimension (mm)	Quantity/Pieces
Dumbbell	Uniaxial tensile test	*l*_1_ = 25; *l*_2_ = 115; *a* = 6; *t* = 0.05	15
Dumbbell	Tensile test with temperature	*l*_1_ = 25; *l*_2_ = 115; *a* = 6; *t* = 0.05	35
Rectangular	Dynamic mechanical analysis	*a* = 6; *b* = 30; *t* = 0.05	10
Square	AFM surface topography	*a* = 6; *t* = 0.05	5

**Table 2:** Electron irradiation arrangements.

Flux	Electron Fluence (e/cm^2^)	Quantity/Pieces		
		Dumbbell	Rectangular	Square
1 × 10^12^ e/(cm^2^·s)	5 × 10^14^	3	2	1
	1 × 10^15^	3	2	1
	5 × 10^15^	3	2	1
	1 × 10^16^	3	2	1

## Data Availability

The data presented in this study are available on reasonable request from the corresponding author.

## Appendix A

After recording the uniaxial tensile test process of the PI film in real time, a DIC algorithm such as Ncorr [34] was taken to analyze the tensile process. Meantime, commercial software ABAQUS (version 6.14) was used to simulate the uniaxial tensile process of a PI specimen. The PI specimen was set as an isotropic elastic material with Poisson’s ratio of 0.34 and Young’s modulus of 2.45 GPa, measured from the experiment. The geometry dimensions of the dumbbell specimen are the same as those in Figure 1a and Table 1. A number of 18,150 8-node linear brick elements with reduced integration and hourglass control (C3D8R) were employed for the PI specimen in the simulation. While one end of the dumbbell specimen was fixed, the other end was subjected to the measured maximum displacement just before elongation at break. Finally, a comparison of the DIC results and FEM simulation is shown in Figure A1. The width of the PI film becomes narrow after elongation, this is also confirmed by the simulation, see Figure A1a. According to Figure A1b, the maximum strain along the stretch direction happened in the middle section of the PI specimen. However, from the DIC results on the left, there is an area with the darkest color, at which point, finally, the PI film breaks.

Three different square areas of 1 μm × 1 μm from the pristine and irradiated PI surfaces were randomly selected for an AFM scan. The measured roughness and calculated average roughness (mean Ra) are listed in Table A1. The standard deviations σ of AFM roughness can be calculated by the following formula:

(A1) $$\sigma =\sqrt{\frac{1}{V\_npnts-1}\Sigma {\left(Y_{i}-V\_avg\right)}^{2}}$$

where V_npnts represents the number of points, V_avg denotes the mean Ra, and $Y_{i}$ is Ra of each area.

**Table A1:** Measured average roughness of pristine and irradiated PI.

	Pristine	5 × 10^14^ e/cm^2^	1 × 10^15^ e/cm^2^	5 × 10^15^ e/cm^2^	1 × 10^16^ e/cm^2^
Area 1	2.606 nm	2.375 nm	2.758 nm	2.14 nm	3.05 nm
Area 2	2.646 nm	2.132 nm	2.931 nm	1.88 nm	2.559 nm
Area 3	2.556 nm	2.447 nm	2.521 nm	2.334 nm	2.538 nm
Mean Ra	2.603 nm	2.318 nm	2.737 nm	2.118 nm	2.716 nm
σ	0.045094346	0.165054536	0.205831242	0.227798156	0.289731773

After the FTIR test of PI with or without electron irradiation, a noticeable difference exists on each peak of different specimens in the transmittance mode, as shown in Figure A2. Based on a previous study [15], the peak observed at 2932 cm^−1^ represents the C-H bonds in tert-butyl and cyclohexyl groups. The peaks around 1775 cm^−1^ and 1718 cm^−1^ represent coupled stretching vibration of carbonyl group C=O. The wavenumber of 1500 cm^−1^ represents stretching vibration band of phenyl ring C=C. Here, 1375 cm^−1^ represents stretching vibration band of amide group =C-N, and 1242 cm^−1^ represents stretching vibration band of aromatic oxide C-O-C. More details are presented in Table A2. However, the test results reveal that fluctuation does not simply increase with irradiation fluence. Finally, the wavenumber presents a most noticeable increase after receiving electron irradiation of 1 × 10^16^ e/cm^2^. Each band mentioned above shows a remarkable difference. When the radiation fluence reaches 1 × 10^16^ e/cm^2^, the chemical bond is more likely to break and cannot recombine, which in consequence causes a fluctuation of the vibration peak in the FTIR spectra. No new characterizing peak is created, meaning no new substance is created after electron irradiation. Chemical bond breakage and recombining of radicals are the most potential reasons for the degradation of mechanical properties and a change in glass transition temperature.

**Table A2:** Spectrum bands at different wavenumbers of pristine and irradiated PI.

Wavenumbers	Pristine	5 × 10^14^ e/cm^2^	1 × 10^15^ e/cm^2^	5 × 10^15^ e/cm^2^	1 × 10^16^ e/cm^2^
2932 cm^−1^	97.2	97.3	97.4	97.2	98.3
1775 cm^−1^	67.5	73.3	68.4	68.4	83.3
1718 cm^−1^	15.0	26.5	15.7	16.6	52.0
1500 cm^−1^	11.8	23.6	13.2	14.0	49.5
1375 cm^−1^	16.7	27.5	18.1	18.7	50.9
1242 cm^−1^	13.5	23.3	14.9	15.5	46.8

Figure A3 shows a thin-film solar cell based on a PI film from Shanghai Institute of Space Power-Sources. The thin-film GaAs solar cell is an inverted metamorphic triple junction (3J IMM) solar cell, with InGaAs, GaAs, and GaInP as the bottom, middle, and top junctions. A bonding metal layer connects the flexible PI substrate and epitaxial layer of the battery. The thickness of the PI substrate is about 50 microns, and the total thickness of the solar cell is about 70 to 80 microns. The influences of radiation and temperature will be investigated in future work.

## References

[1] Wang K., Wang X., Qian B., Ma J., Lu J. (2022). Recent Development OnSpace Application for High-Efficiency Solar Cells and Array Technology. Kuei Suan Jen Hsueh Pao/J. Chin. Ceram. Soc..

[2] Zhang F., Han W., Chen H., He H., Wang X. (2021). The Design of Flexible Printed Circuit in Solar Cell Array and Experimental Study of Its Thermal Adaptability. Spacecr. Environ. Eng..

[3] Yin M.S., Yang G., Wang X.C., Fan B., Jiang D.P., Yang H.D. (2021). Strain-Testing Research of Space Solar Cell Array. Wuli Xuebao/Acta Phys. Sin..

[4] Miller S.K.R., Dever J.A. Space Environment Exposure Results from the MISSE 5 Polymer Film Thermal Control Experiment on the International Space Station. Proceedings of the 11th International Symposium on Materials in the Space Environment.

[5] Miller S.K.R., Dever J.A., Banks B.A., Kline S. MISSE 6 Polymer Film Tensile Experiment. Proceedings of the 2010 National Space and Missile Materials Symposium (NSMMS).

[6] Miller S.K.R., Banks B.A., Sechkar E. An Investigation of Stress Dependent Atomic Oxygen Erosion of Black Kapton Observed on MISSE 6. Proceedings of the 2010 National Space and Missile Materials Symposium (NSMMS).

[7] Miller S.K.R., Sechkar E.A. (2013). An Examination of Radiation Induced Tensile Failure of Stressed and Unstressed Polymer Films Flown on MISSE 6. https://ntrs.nasa.gov/api/citations/20130001603/downloads/20130001603.pdf.

[8] Shimamura H., Nakamura T. (2010). Investigation of Degradation Mechanisms in Mechanical Properties of Polyimide Films Exposed to a Low Earth Orbit Environment. Polym. Degrad. Stab..

[9] Shimamura H., Yamagata I. (2012). Degradation of Mechanical Properties of Polyimide Film Exposed to Space Environment. J. Spacecr. Rocket..

[10] Shen Z., Liang G., Ziliang M., Yu B., Yigang D., Yenan L., Zhihao W. (2016). Mechanical Property Degradation of Polyimide Film under Gamma Ray Radiation. Spacecr. Environ. Eng..

[11] Dong S.S., Shao W.Z., Yang L., Ye H.J., Zhen L. (2018). Surface Characterization and Degradation Behavior of Polyimide Films Induced by Coupling Irradiation Treatment. RSC Adv..

[12] Dong S.S., Shao W.Z., Yang L., Ye H.J., Zhen L. (2018). Microstructure Evolution of Polyimide Films Induced by Electron Beam Irradiation-Load Coupling Treatment. Polym. Degrad. Stab..

[13] Manas D., Mizera A., Manas M., Ovsik M., Hylova L., Sehnalek S., Stoklasek P. (2018). Mechanical Properties Changes of Irradiated Thermoplastic Elastomer. Polymers.

[14] Manas D., Ovsik M., Mizera A., Manas M., Hylova L., Bednarik M., Stanek M. (2018). The Effect of Irradiation on Mechanical and Thermal Properties of Selected Types of Polymers. Polymers.

[15] Stelescu M.D., Airinei A., Manaila E., Craciun G., Fifere N., Varganici C., Pamfil D., Doroftei F. (2018). Effects of Electron Beam Irradiation on the Mechanical, Thermal, and Surface Properties of Some EPDM/Butyl Rubber Composites. Polymers.

[16] Qiu J., Heini M., Ma J., Han W., Wang X., Yin J., Shi Y., Gao C. (2023). Mechanical Properties of Multi-Scale Germanium Specimens from Space Solar Cells under Electron Irradiation. Chin. J. Aeronaut..

[17] Plis E.A., Engelhart D.P., Cooper R., Johnston W.R., Ferguson D., Hoffmann R. (2019). Review of Radiation-Induced Effects in Polyimide. Appl. Sci..

[18] Zhang H., Liu H.W., Hu Z.Y., Zhang W. (2019). On-Orbit Load Analysis of Solar Wing with Flexible Characteristics. Yuhang Xuebao/J. Astronaut..

[19] Sibin K.P., Mary Esther A.C., Shashikala H.D., Dey A., Sridhara N., Sharma A.K., Barshilia H.C. (2018). Environmental Stability of Transparent and Conducting ITO Thin Films Coated on Flexible FEP and Kapton^®^ Substrates for Spacecraft Applications. Sol. Energy Mater. Sol. Cells.

[20] Cherkashina N.I., Pavlenko V.I., Noskov A.V. (2019). Synthesis and Property Evaluations of Highly Filled Polyimide Composites under Thermal Cycling Conditions from −190 °C to +200 °C. Cryogenics.

[21] Kang P.H., Jeon Y.K., Jeun J.P., Shin J.W., Nho Y.C. (2008). Effect of Electron Beam Irradiation on Polyimide Film. J. Ind. Eng. Chem..

[22] Mishra R., Tripathy S.P., Dwivedi K.K., Khathing D.T., Ghosh S., Müller M., Fink D. (2003). Spectroscopic and Thermal Studies of Electron Irradiated Polyimide. Radiat. Meas..

[23] Sasuga T. (1991). Electron Irradiation Effects on Dynamic Viscoelastic Properties and Crystallization Behaviour of Aromatic Polyimides. Polymer.

[24] Hirade T., Hama Y., Sasuga T., Seguchi T. (1991). Radiation Effect of Aromatic Thermoplastic Polyimide (New-TPI). Polymer.

[25] Kupchishin A.I., Muradov A.D., Omarbekova Z.A., Taipova B.G. (2007). Mechanooptical Investigations of Polyimide Films Exposed to Electrons, Mechanical Loads, and Temperatures. Russ. Phys. J..

[26] (2018). Space Systems—Space Solar Cells—Electron and Proton Irradiation Test Methods. https://www.iso.org/standard/69495.html.

[27] (2019). China, S.A. of Test Method of Electron Irradiation Aerospace Solar Cells. http://c.gb688.cn/bzgk/gb/showGb?type=online&hcno=B3F21AA1A502A459C4E7208AD380B3B8.

[28] (2006). China, S.A. of Plastics—Determination of Tensile Properties—Part 3: Test Conditions for Films and Sheets. https://openstd.samr.gov.cn/bzgk/gb/newGbInfo?hcno=39C9E88D2852DFAF876475C8AB7A97E2.

[29] Rahnamoun A., Engelhart D.P., Humagain S., Koerner H., Plis E., Kennedy W.J., Cooper R., Greenbaum S.G., Hoffmann R., van Duin A.C.T. (2019). Chemical Dynamics Characteristics of Kapton Polyimide Damaged by Electron Beam Irradiation. Polymer.

[30] Ennis C.P., Kaiser R.I. (2010). Mechanistical Studies on the Electron-Induced Degradation of Polymethylmethacrylate and Kapton. Phys. Chem. Chem. Phys..

[31] Zhao F., Zhang H., Zhang D., Wang X., Wang D., Zhang J., Cheng J., Gao F. (2022). Molecular Insights into the ‘Defects’ Network in the Thermosets and the Influence on the Mechanical Performance. RSC Adv..

[32] Plis E.A., Bengtson M.T., Engelhart D.P., Badura G.P., Cowardin H.M., Reyes J.A., Hoffmann R.C., Sokolovskiy A., Ferguson D.C., Shah J.R. (2022). Characterization of Novel Spacecraft Materials under High Energy Electron and Atomic Oxygen Exposure. Proceedings of the AIAA Science and Technology Forum and Exposition, AIAA SciTech Forum 2022.

[33] Cherkashina N.I., Pavlenko V.I., Noskov A.V., Romanyuk D.S., Sidelnikov R.V., Kashibadze N.V. (2022). Effect of Electron Irradiation on Polyimide Composites Based on Track Membranes for Space Systems. Adv. Sp. Res..

[34] Blaber J., Adair B., Antoniou A. (2015). Ncorr: Open-Source 2D Digital Image Correlation Matlab Software. Exp. Mech..

